# A Case of Deep Vein Thrombosis After Recovery From COVID-19 and Its Association With Elevated D-dimers

**DOI:** 10.7759/cureus.29859

**Published:** 2022-10-03

**Authors:** Naresh Dasari, Akshat Banga, Namratha Pallipamu, Trupti Pandit, Ramesh Pandit, Ramesh Adhikari

**Affiliations:** 1 Internal Medicine, Roger Williams Medical Center, Providence, USA; 2 Internal Medicine, Sawai Man Singh Medical College, Jaipur, IND; 3 Hospital Medicine, Franciscan Health, Lafayette, USA; 4 Pediatrics, Nemours Children's Health, Glen Mills, USA; 5 Medicine, Independent Researcher, Philadelphia, USA; 6 Hospital Medicine, University of Pennsylvania/Chester County Hospital, Philadelphia, USA; 7 Geriatrics, Brown University, Providence, USA

**Keywords:** long-term, thromboembolic disease, coronavirus disease, d-dimer, covid-19 pandemic, hypercoagulable, post-covid-19, covid-19 recovery, deep vein thrombosis (dvt), covid-19

## Abstract

The coronavirus disease 2019 (COVID-19) continues to be a devastating disease for the elderly population, especially in long-term care facilities, and it presents with varying clinical presentations. We have ample evidence that COVID-19 can predispose to deep vein thrombosis (DVT) and pulmonary embolism (PE) during an active infection. Still, very few cases of DVT have been reported after recovery from COVID-19.

The imbalance of the coagulation cascade and the increased release of certain coagulation factors play an essential role in promoting hypercoagulability and vascular endothelial dysfunction. It leads to a rise in the level of fibrin degradation products, D-dimers, which can remain elevated for up to several weeks, even after recovery. It has been suggested that the risk of DVT occurring after recovering from COVID-19 remains high for up to three months.

We report a case of a 77-year-old long-term care female resident at a nursing facility, ambulatory at baseline, who was noted to be COVID-19 positive upon routine facility-wide testing per department of health guidelines. She was asymptomatic during her 10-day quarantine period. D-dimer levels during routine labs were high (initial D-dimer level of 1.87 mg/L FEU {normal value: 0.19-0.52 mg/L FEU}), but the patient had no clinical signs and symptoms of DVT. Ultrasound of the bilateral legs was not performed due to low clinical suspicion. The patient received an enoxaparin DVT prophylaxis dose during the quarantine period. Follow-up D-dimer levels were done at frequent intervals after recovery, but D-dimer levels continued to remain elevated up till six weeks after her 10-day quarantine period ended. Based on previous experience with other long-term care residents who suffered from COVID-19, bilateral lower extremity ultrasound was performed, which showed bilateral DVT.

Elevated D-dimer levels are a predictor of hypercoagulation complications in COVID-19. Patients with persistently elevated D-dimer levels after recovery from COVID-19 should be screened for thromboembolic complications, even if they are asymptomatic. DVT can occur up to three months post-recovery from COVID-19 infection.

## Introduction

Severe acute respiratory syndrome coronavirus 2 (SARS-CoV-2) was discovered in Wuhan, China, in the epidemic of pneumonia which started in December 2019 and spread rapidly all over the world and became a global pandemic [1]. In February 2020, the World health organization (WHO) named the disease coronavirus disease 2019 (COVID-19) [2]. The initial clinical presentations of COVID-19 were similar to any other flu, including fever, cough, breathing difficulties, and pneumonia, which further progressed to acute respiratory disease involving alveolar damage and even death due to multiorgan failure [3,4]. In addition, hypercoagulability has been a concerning complication in COVID-19 patients and those recovering from it [5,6]. In a meta-analysis conducted on COVID-19 patients by Suh et al., it was shown that the pooled incidence rate of pulmonary embolism (PE) was 16.5%, and deep vein thrombosis (DVT) was 14.8% [7]. It was understood that viral infection-mediated inflammation is associated with pro-thrombotic changes, including the fibrin clot dynamics and turbidity. It likely causes elevated fibrinogen and D-dimer levels [8].

## Case presentation

A 77-year-old long-term care female resident at the nursing facility with a past medical history of type 2 diabetes mellitus, asthma, alcoholic liver cirrhosis, peripheral vascular disease, coronary artery disease, gastroesophageal reflux disease (GERD), anemia, benign hypertension, history of chest tube insertion, and cholecystectomy. The patient was ambulatory at baseline and had no personal history of any malignancy. No family history of hypercoagulable disease or thromboembolism was present. She had a remote smoking history and no alcoholism or substance abuse history. Current medications include acetaminophen, artificial tears, aspirin, calcium, Lantus, losartan, multivitamins, omeprazole, Senna Plus, simvastatin, trazodone, and vitamin D supplements.

**Figure 1:**
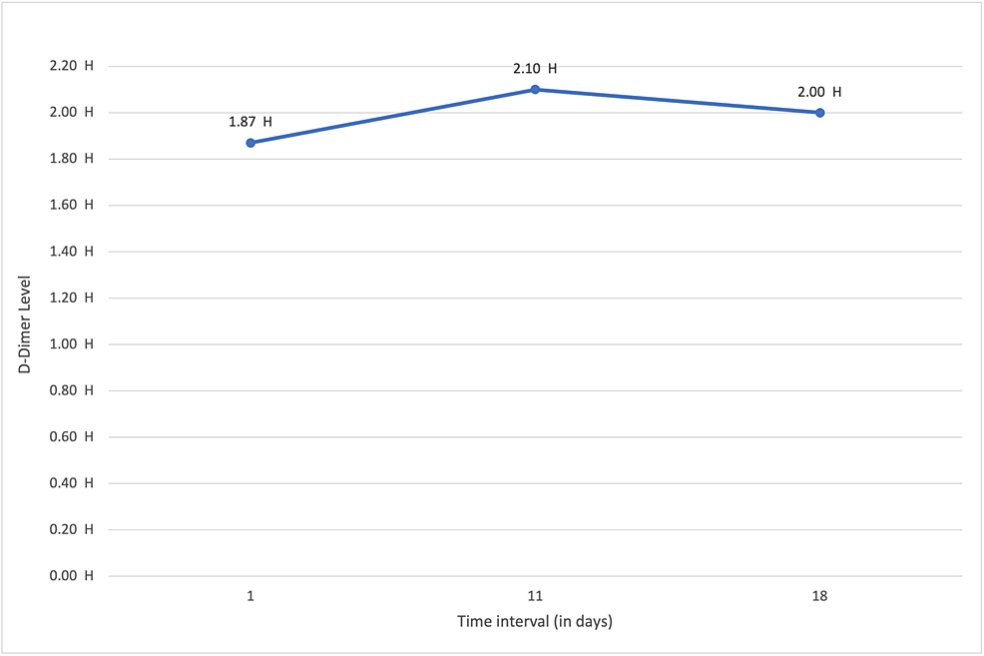
D-dimer level on consecutive follow-up visits after recovery from COVID-19. COVID-19: coronavirus disease 2019

The patient tested positive for COVID-19 in routine facility-wide testing per Department of Health guidelines. The patient denied fever, cough, chest pain, or lower-extremity edema. She was asymptomatic during her 10-day quarantine period. D-Dimer levels were high upon initial blood work, but the patient had no clinical signs and symptoms of DVT. The patient received enoxaparin for DVT prophylaxis. Follow-up D-dimer levels were done at frequent intervals even after recovery, but the D-dimer levels continued to remain high even after the patient was off her 10-day quarantine period (Table 1 and Figure 1). Pertinent findings on physical examination included clear breath sounds and a regular, rapid heart rhythm on auscultation. There was no lower-extremity edema or calf tenderness. After she completed her 10-day quarantine period, she was off the COVID-19 isolation precautions, but her D-dimer levels continued to be elevated for the following several weeks. Based on previous experience with other long-term care residents, bilateral lower extremity ultrasound was performed six weeks after a quarantine period of 10 days, which demonstrated - acute non-occlusive DVT within the right profunda and left mid-femoral veins.

**Table 1:** Follow-up D-dimer levels after COVID-19 recovery. COVID-19: coronavirus disease 2019

Time interval	D-dimer level	Reference value
Initial	1.87 mg/L FEU	0.19-0.52 mg/L FEU
11 days later	2.10 mg/L FEU	0.19-0.52 mg/L FEU
18 days later	2.00 mg/L FEU	0.19-0.52 mg/L FEU

The patient was treated with apixaban 10 mg PO twice daily for the first 10 days, followed by 5 mg PO bid. At the time of this report, she continued to be on apixaban and planned for a follow-up ultrasound of the lower extremities. According to the laboratory data, complete metabolic panel is within normal limits. Complete blood count with differential is within normal limits.

## Discussion

Patients with COVID-19 and multiple co-morbidities are susceptible to hypercoagulability [9,10]. An observational study by Katsoularis et al. suggested that COVID-19 is an independent risk factor for DVT, and a higher DVT risk persists for up to three months post-recovery [10]. Although thromboembolic complications have been well documented in COVID-19, cases of DVT occurring after recovery from the viral infection, along with persistent elevation of D-dimer, have been rarely reported [11]. Our case report describes the occurrence of DVT in an elderly female six weeks into her recovery from asymptomatic COVID-19. Doppler ultrasound of bilateral lower limbs showed non-occlusive DVT in an ambulatory patient post-COVID-19.

In other studies of COVID-19 patients, Hesam-Shariati et al. and Debela et al. reported patients presenting with typical symptoms of DVT involving leg swelling, redness, calf tenderness, and decreased limb mobility [12,13]. While our patient was clinically asymptomatic, DVT was suspected based on persistently elevated D-dimer levels for several weeks post-recovery and confirmed by Doppler ultrasonography.

The SARS-CoV-2 virus can cause coagulopathy in multiple ways, including direct vascular endothelial injury and stimulation of an inflammatory cascade [9]. Vascular endothelial cells are essential in regulating vascular permeability and maintaining hemostasis. While endothelial dysfunction can disrupt vascular hemostasis, the virus can also enter a host cell and stimulate the secretion of proinflammatory cytokines and antifibrinolytic chemokines, promoting a hyperinflammatory condition that can lead to acute respiratory distress syndrome (ARDS), acute cardiac injury, acute kidney injury, septic shock, and death [14]. Hypercoagulability in COVID-19 is linked to vascular endothelial dysfunction, hyperinflammation, and coagulation cascade activation, including increased von Willebrand factor (VWF), factor VIII, fibrinogen, and hyperviscosity [15]. Breakdown of the thrombi formed leads to elevation of D-dimer levels and fibrin degradation product levels [14]. A retrospective multicenter cohort study found that patients who died from COVID-19 had a higher probability of having leukocytopenia, elevated D-dimers, proinflammatory cytokines like interleukin 6 (IL-6), interferon‐gamma (IFN‐γ), and lactate dehydrogenase (LDH), and prolonged prothrombin time (PT) [16,17].

It is vital to monitor coagulation function in hospitalized COVID-19 patients through repeated measurements of D-dimer levels. Although the change in D-dimer levels can predict the severity of hypercoagulation complications, the level of COVID-19 disease severity in patients admitted to the intensive care unit (ICU), and their potential mortality remains unclear [18,19]. Studies have shown that serum D-dimer levels can be a helpful tool for risk stratification in COVID-19 infection, along with being reliable in predicting thromboembolic complications [18,19]. In a single center-based cohort study, Middeldorp et al. found that COVID-19 patients admitted to the ICU had significantly raised D-dimer levels on admission compared to non-ICU patients, even after receiving prophylactic anticoagulation [20]. In our case report, we found that the patient had increased D-dimer levels, not only during the active infection period but also post-recovery, and it was, in turn, linked to an increased risk of DVT.

The WHO approved prophylactic low molecular weight heparin (LMWH) enoxaparin for venous thromboembolism in COVID-19-associated hypercoagulation management [21]. Along with its anticoagulant properties, LMWH has shown some antiinflammatory attributes, which might help alleviate the inflammatory response caused by the SARS-CoV-2 virus. Heparin suppresses IL-6 and IL-8 expression in lung epithelial cells, reducing the risks of thromboembolic complications and proinflammatory cytokine storms [22]. In this case, the patient received a DVT prophylaxis dose of enoxaparin, but it did not prevent the development of DVT in her post-COVID time.

## Conclusions

COVID-19 infection continues to cause significant morbidity and mortality in elderly patients. Hypercoagulable complications due to COVID-19 are mainly reported during the active infection but can present up to three months after complete recovery from the disease. DVT in elderly populations post-COVID-19 infection can be clinically asymptomatic. However, it should be considered in the differential diagnosis if D-dimers are persistently elevated or has clinical symptoms.
